# Unraveling genetics of semi-determinacy and identification of markers for indeterminate stem growth habit in chickpea (*Cicer arietinum* L.)

**DOI:** 10.1038/s41598-021-01464-3

**Published:** 2021-11-08

**Authors:** Ambika, Venkatraman Hegde, M. S. Nimmy, C. Bharadwaj, Shailesh Tripathi, Rajesh Kumar Singh, Rajendra Kumar

**Affiliations:** 1 Division of Genetics, ICAR-Indian Agricultural Research Institute, New Delhi, 110 012 India; 2 ICAR-National Institute for Plant Biotechnology, New Delhi, 110 012 India

**Keywords:** Agricultural genetics, Plant breeding, Plant genetics

## Abstract

Chickpea (*Cicer arietinum* L.) is predominantly an indeterminate plant and tends to generate vegetative growth when the ambient is conducive for soil moisture, temperature and certain other environmental conditions. The semi-determinate (SDT) types are comparatively early, resistant to lodging and found to be similar in their yield potential to indeterminate (IDT) lines. Indeterminate and semi-determinate genotypes are found to be similar during early stage, which makes it difficult to distinguish between them. Thus, there is a need to identify molecular markers linked either to indeterminate or semi-determinate plant types. The present study was carried out to study the genetics of semi-determinacy and identify molecular markers linked to stem growth habit. The study was undertaken in the cross involving BG 362(IDT) × BG 3078-1(SDT). All F_1_ plants were indeterminate, which indicates that indeterminate stem type is dominant over semi-determinate. In further advancement to F_2_ generation, F_2_ plants are segregated in the ratio of 3(Indeterminate): 1(Semi-determinate) that indicates that the IDT and SDT parents which are involved in the cross differed for a single gene. The segregation pattern observed in F_2_ is confirmed in F_3_ generation. The parental polymorphic survey was undertaken for molecular analysis using total of 245 SSR markers, out of which 41 polymorphic markers were found to distinguish the parents and were utilized for bulked segregant analysis (BSA). The segregation pattern in F_2_ indicates that the IDT (Indeterminate) and SDT (Semi-determinate) parents which are involved in the cross differed for single gene. The segregation pattern of F_2_ and F_3_ derived from the cross BG 362 (IDT) × BG 3078-1 (SDT) confirmed the genotypic structure of the newly found SDT genotype BG 3078-1 as *dt1dt1Dt2Dt2*. Three SSR markers *TA42*, *Ca_GPSSR00560* and *H3DO5* were found to be putatively linked to *Dt1* locus regulating IDT stem growth habit. Our results indicate that the SSR markers identified for *Dt1* locus helps to differentiate stem growth habit of chickpea in its early growth stage itself and can be efficiently utilized in Marker Assisted Selection (MAS) for changed plant type in chickpea.

## Introduction

Chickpea (*Cicer arietinum* L.) is an annual diploid (2n = 2x = 16) legume crop with a genome size of 738 Mb with 28,269 genes^1^. The plants can be determinate (DT), semi-determinate (SDT) and indeterminate (IDT). Indeterminate types are characterized by vegetative buds at terminal meristems and stem apices, which regulate the development of new nodes with leaves and produce inflorescence in axillary meristem and therefore, the stem keeps on growing in length and keeps producing flowers and pods till temperature and humidity allows^2,3^. The plants with semi-determinate growth habit are similar to an indeterminate but each terminal meristem carry a floral bud, which terminates further growth. In determinate types, the terminal meristems transformed from a vegetative state to a reproductive phase leading to the development of a terminal flower and as a consequence, the vegetative growth stops^2,4^. Therefore, the stem growth habit plays a significant role in determining plant architecture, which is of major agronomic importance and specifies plant adaptability to crop cultivation as well as seed yield potential^5^. The modification of plant architecture improves crop adaptation to various environments and enhances the yield including its stability^6^.

Chickpea is predominantly an indeterminate type and tends to generate a vegetative phase, when the temperature, soil moisture and certain other environmental conditions are conducive^7^. Due to its indeterminate habit, excessive water induces vegetative development, which serves as a competitive sink for pod formation while reduces the fruit set^8^. The indeterminate growth allows competition between the vegetative and reproductive phases for assimilation partitioning. This promotes low and unstable harvest index leading to low seed yield. It causes considerably long cycles and late maturation, due to the prolonged vegetative phase^6^. Chickpea is primarily grown under marginal conditions in rainfed areas predominated by lack of moisture and fertility. Therefore, alteration of plant architecture from indeterminate to semi-determinate or determinate type will restrict vegetative growth and improve productivity and stabilization of production.

The stem growth habit in chickpea is known to be controlled by two genes (*Dt1/dt1* and *Dt2/dt2*) with dominance epistasis^9^. *Dt1* allele is epistatic to both *Dt2* and *dt2* alleles. The indeterminate plants carry *Dt1* allele either in homozygous (*Dt1Dt1Dt2*- and *Dt1Dt1dt2dt2*) or heterozygous (*Dt1dt1Dt2*- and *Dt1dt1dt2dt2*) condition. The semi-determinate plants are homozygous recessive for *dt1* and homozygous or heterozygous for *Dt2* (*dt1dt1Dt2Dt2*, *dt1dt1Dt2dt2*). The determinate plants carry recessive alleles at both loci in homozygous condition (*dt1dt1dt2dt2*)^9^.

The performance of the crop is adversely affected by high fertility and irrigation. SDT types are comparatively early, lodging resistant and found to be similar in their yield potential to that of IDT types. A SDT mutant was more responsive to supplemental nitrogen as compared to its IDT parent^10^. A change in plant type from IDT to SDT or DT is therefore required to improve the adaptation of chickpea plants to better agronomy and cool climate to achieve a breakthrough in its productivity. A better understanding of the inheritance of SDT growth habit would facilitate the breeding of chickpea cultivars that would be better responsive to the cool climate and more productive environments. During early vegetative growth, the SDT plants are similar in their stem growth habit to IDT plants and often it is difficult to distinguish indeterminate or semi-determinate chickpeas in breeding populations. Marker-assisted selection (MAS) has huge potential to enhance selection efficiency in chickpea breeding programs for alternative stem types. Hence, the development of molecular markers associated with indeterminate or semi-determinate stem growth would enable one to distinguish plant types for selection in the seedling stage itself. Breeding to improve the plant type of existing cultivars from IDT to DT/SDT stem growth with better response to high input is necessary to achieve an improvement in productivity.

Following were the main objectives of this study:

(i) To confirm and elucidate the genetics of semi-determinant growth habit, and
(ii) To identify molecular markers linked to stem growth habit using bulked segregant analysis (BSA).

## Results

**Figure 1:**
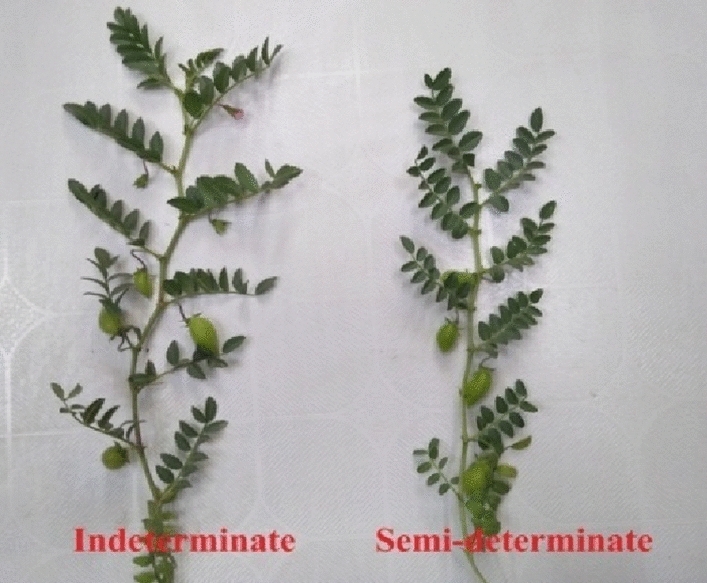
Stem growth habit of BG362 (IDT) and BG3078-1(SDT) at early stage.

The two parents (SDT BG 3078-1 and IDT, BG 362) did not differ except the branch termination pattern. The branches of BG 3078-1 terminated by a flower bud or a fully opened flower (Fig. 1). The SDT genotype BG 3078-1 flowered in 56 days and matured in 119 DAS whereas the IDT genotype BG 362 (IDT) flowered in 75 days and reached maturity in 135 DAS^11^.

### Genetics of semi-determinate stem growth habit

All the F_1_ plants were indeterminate suggesting that indeterminate growth habit is dominant over semi-determinate. The 183 F_2_ plants of the cross BG362 × BG3078-1 segregated into 138 indeterminates and 45 semi-determinates (Table 1). These data gave a good fit to the ratio of 3 indeterminate: 1 semi-determinate stem growth habit (χ^2^ = 0.029, P = 0.9–0.5) indicating that the IDT and SDT parents involved in the cross differed for a single gene *Dt1*, which is known to control the IDT phenotype and is epistatic over *Dt2*.

**Table 1:** Segregation for stem growth habit in F_2_ of a chickpea cross involving indeterminate and semi-determinate parents.

Cross	Total plants	Observed		Expected		Ratio tested	χ^2^ value	P value
		IDT	SDT	IDT	SDT			
BG 362 × BG 3078-1								
BG362	1	1	0					
BG 3078-1	1	0	1					
F_1_	1	1	0					
F_2_	183	138	45	137	46	3:1	0.029	0.9–0.5

The data of segregating and non-segregating progenies in F_2:3_ are presented in Table 2, which suggested monogenic segregation. All 43 F_2_ semi-determinate plants bred true in F_2:3_ . Out of the 129 progenies raised from indeterminate F_2_ plants, 96 segregated into indeterminate and semi-determinate plants and 33 did not segregate. The proportion of non-segregating and segregating progenies recorded in F_2:3_ indeterminate plants gave a good fit to the expected pattern 2 segregating: 1 non-segregating progenies (χ^2^ = 3.48, P = 0.1–0.05). Thus, the segregation patterns of F_2_ and F_2:3_ were in agreement with the known SDT genotype, thus confirming that the genotype of BG 3078-1 is *dt1dt1Dt2Dt2*. The descriptive statistics of quantitative characters of F_2_ population are presented in the Supplementary Table S3.

**Table 2:** Segregation for stem growth habit in F_3_ of a chickpea cross involving indeterminate and semi-determinate parents.

Cross	Phenotypic class	No. of progenies	Observed		Expected		Ratio tested	χ^2^ value	P value
			Segregating	Non-segregating	Segregating	Non-segregating			
BG362 × BG3078-1	IDT	129	96	33	86	43	2:1	3.48	0.1–0.05
	SDT	43	0	43	0	43	0:1	0.00	1.00

*IDT* indeterminate, *SDT* semi-determinate.

### Molecular markers linked to genes for stem growth habit using BSA

#### Parental polymorphic survey

**Figure 2:**
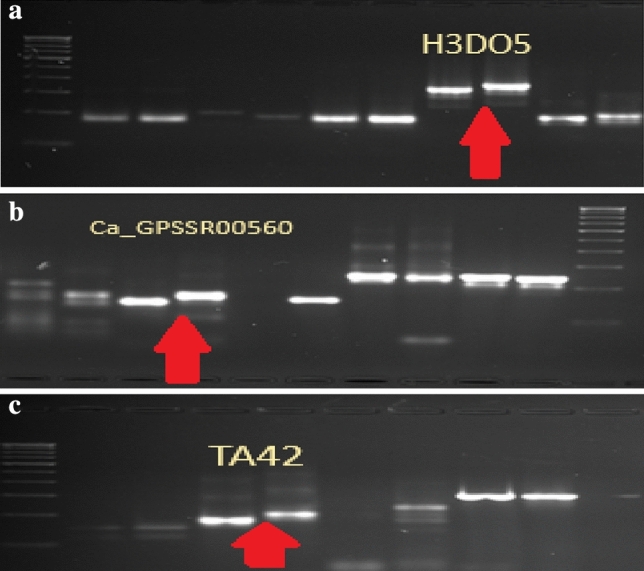
(**a**) Parental polymorphism of *H3DO5* between BG362 (IDT) and BG3078-1(SDT). (**b**) Parental polymorphism of *Ca_GPSSR00560* between BG362 (IDT) and BG3078-1(SDT). (**c**) Parental polymorphism of *TA42* between BG362 (IDT) and BG3078-1(SDT).

The parental polymorphic survey was conducted as detailed in Material and Methods; 41 SSR markers were polymorphic between the two parents involved in the cross. The gel pictures of polymorphic SSR markers between two parents are shown in the Fig. 2.

#### Bulked segregant analysis

**Figure 3:**
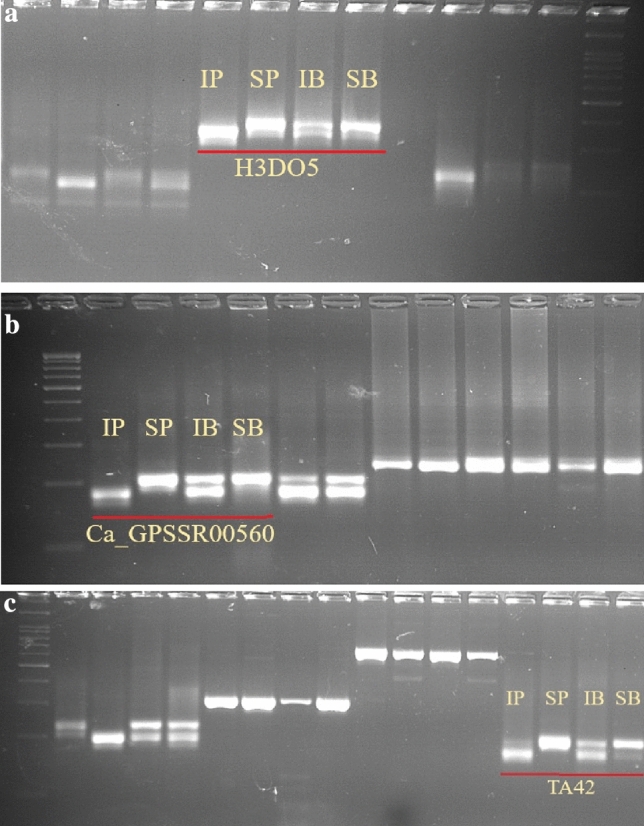
(**a**) Bulked Segregant Analysis (BSA) of *H3DO5* showing putatively linked marker. (**b**) Bulked Segregant Analysis (BSA) of *Ca_GPSSR00560* showing putatively linked marker. (**c**) Bulked Segregant Analysis (BSA) of *TA42* showing putatively linked marker. *IP* indeterminate parent, *SP* semi-determinate parent, *IB* indeterminate bulk, *SB* semi-determinate bulk.

Among 41 polymorphic SSR markers, 3 SSR markers were found to be polymorphic between the two bulks of DNA along with parents (Fig. 3). Three SSR markers, namely *Ca_GpSSR00560, TA42* and *H3DO5* co-segregated with the indeterminate stem growth habit and were therefore accepted to be associated with the gene *Dt1* in chickpea. The marker, *Ca_GpSSR00560* was reported to be located on linkage group 4^12^, *TA42* was mapped on linkage group 7^13^ and *H3DO5* was mapped on linkage group 1^14^.

## Discussion

It is known that stem growth in chickpea is regulated by two genes, namely *Dt1/dt1* and *Dt2/dt2* with dominant allele *Dt1* epistatic to *Dt2* as well as *dt2*^9^. The presence of *Dt1* allele gave indeterminate growth habit, irrespective of the genotype at the other locus, so that indeterminate type could be either homozygous (*Dt1Dt1Dt2-* or *Dt1Dt1dt2dt2*) or heterozygous (*Dt1dt1Dt2*- or *Dt1dt1dt2dt2*). The semi-determinate would have *Dt2* allele either in the homozygous (*dt1dt1Dt2Dt2*) or heterozygous (*dt1dt1Dt2dt2*) state subject to the presence of recessive allele at the other locus (*dt1dt1*). A determinate phenotype would then be homozygous recessive at both the loci (*dt1dt1dt2dt2*). The results of the present study confirmed these genotypes (Tables 1, 2). The results suggested that the genotype IDT parent BG 362 was inferred as *Dt1Dt1Dt2Dt2* and carries *Dt1* and *Dt2* alleles in homozygous condition at both the loci as reported with another inderminate parent BGD 72 in the earlier study^9^. Hence, the genotype of the SDT genotype BG3078-1 is inferred to be as *dt1dt1Dt2Dt2*. The dominance of the IDT stem type has also been reported earlier in chickpea^9,15,16^, *Glycine max*^4^, *Vicia faba*^17^ and *Cajanus cajan*^18,19^.

These results obtained in the present study were similar to the inheritance study conducted earlier in pigeonpea^18^. The segregation pattern of F_2_ and F_2:3_ derived from the cross BG362 (IDT) × BG3078-1(SDT) confirmed/validated the genotypic structure of the SDT genotype BG3078-1 as *dt1dt1Dt2Dt2* as reported in our previous studies on chickpea^15^.

The study of parental polymorphism involving 245 SSR markers that were distributed evenly on all 16 chromosomes of chickpea resulted in identification of 41 polymorphic markers. BSA was conducted to identify putatively linked molecular markers for *Dt1* locus using polymorphic markers. For this purpose, IDT and SDT bulks were prepared separately by mixing equal amounts of DNA from 20 individuals showing indeterminate and 20 semi-determinate F_2_ plants respectively. Among 41 polymorphic markers, 3 markers showed polymorphism between bulks along with two parents *i.e., Ca_GpSSR00560, TA42* and *H3DO5*. These 3 markers namely *Ca_GpSSR00560, TA42* and *H3DO5* are considered as co-segregating with *Dt1* locus and reported to be present in different genomic regions on LG4^12^, LG7^13^ and LG1^14^ respectively. In contrast to earlier reports^9,15^ our study suggests that besides the two non allelic epistatic genes, there must be additional, minor QTL/modifier involved in regulation of the stem growth habit or just by chance, the 20 SDT lines differed at those two extra markers from the 20 IDT lines; or this particular population being a different one facilitated identification of two additional markers. Out of 3 identified linked markers, the marker *TA42* was already reported to be linked with *Dt1* locus in chickpea^15^. Thus, our study validates that the association of *TA42* with the stem growth habit and can be used in marker assisted selection. Similarly in other legume crops, closely linked marker *TA34* for stem growth habit in soybean^20^ and *CcLG03* for IDT growth habit locus (*Dt1*) in pigeonpea^1,21^ have also been identified/mapped. Thus, these three markers *Ca_GpSSR00560, TA42* and *H3DO5* may be considered as putatively linked to the *Dt1* locus in chickpea.

Overall, our data involving segregation pattern in F_2_ and F_3_ derived from BG362 (IDT) × BG3078-1(SDT) confirmed or validated the genotype of SDT line BG3078-1 as *dt1dt1Dt2Dt2*. Molecular analysis revealed that *TA42, H3DO5* and *Ca_GpSSR00560* SSR markers are putatively linked to *Dt1* locus in chickpea. Our study validated that the association of *TA42* with the stem growth habit and can be used in MAS. Since, the other two markers, *Ca_GpSSR00560* and *H3DO5* are located on LG4 and LG1 respectively, a further detailed investigation is required to confirm their association with the stem growth habit in chickpea. Further investigation on mapping of *Dt1* and *Dt2* loci is required for locating the exact genomic region involved in the inheritance of stem growth habit in chickpea. The markers identified for the *Dt1* locus helps to differentiate the stem growth habit of chickpea in its early growth stage itself and can be efficiently utilized in MAS for changed plant type in chickpea.

## Methods

### Experimental plots

The experimental research and field studies on chickpea were carried out using a cross involving ICAR-IARI developed varieties/germplasm BG362 (IDT) and BG3078-1(SDT) following the national guidelines and legislation in an un-replicated design in the experimental field of Division of Genetics, IARI, New Delhi. This area is located between latitude 28.61° N and longitude 77.23° E and found at an altitude of 225 m above mean sea level. The topography of the experimental plot was uniform.

In 2016–2017 and 2018–2019, research was carried out in MB-5C field plot of Division of Genetics. The soil was sandy loam with physical and nutritional compositions as follows: The pH of soil was alkaline about 8.5–9.2 with low EC about 0.4–0.6 dS/m, low organic content (< 0.5%), low nitrogen (< 280 kg/ha), high phosphorous (24–50 kg/ha) and high potassium (> 280 kg/ha), medium sulphur (10–20 mg/kg), adequate zinc (1–5 mg/kg), adequate iron (5.8–10 mg/kg), adequate manganese (10–25 mg/kg) and adequate copper (0.5–10 mg/kg) respectively.

In 2017–2018 and 2019–2020, studies were undertaken in MB-3A experimental plot of Division of Genetics. The soil was sandy loam with physical and nutritional compositions as follows: The pH of soil was mild alkaline (7.5–8.5) with low EC (0.4–0.6 dS/m), low organic content (< 0.5%), low nitrogen (< 280 kg/ha), high phosphorous (24–50 kg/ha) and high potassium (> 280 kg/ha), medium sulphur (10–20 mg/kg), adequate zinc (1–5 mg/kg), deficient iron (2.5–5.8 mg/kg) adequate manganese (10–25 mg/kg) and adequate copper (0.5–10 mg/kg) respectively.

### Plant materials

A cross was made between the parents BG362 (IDT) and BG3078-1 (SDT) during the *rabi*, the post rainy season of 2016–2017 (morphological characteristics of parents are listed in supplementary Table S2). During *rabi* 2016–2017, the mean daily minimum and maximum temperatures were 10.6 °C and 26.3 °C respectively. The rainfall in the area ranged from 0.5 mm to 57.4 mm with a mean of 0.6 mm with average relative humidity (87.34%).

The parents and F_1_s were planted during the *rabi* season of 2017–2018 and F_1_s were selfed to obtain the F_2_ generation seeds. The environmental condition during the season included the mean daily minimum and maximum temperature as 9.5 °C and 26.5 °C respectively, rainfall ranging from 2.6 mm to 8.6 mm with a mean of 0.165 mm and average relative humidity (82.69%).

The parents and the F_2_ seeds were grown during the *rabi* 2018–2019 to raise F_2_ mapping population. Twenty seeds of each of the parents were sown in each row of 4 m length and 183 F_2_ seeds were sown in 15 rows of 4 m length with a maximum of 13 plants/row. The crop was provided optimum basal dosage of fertilizer N (20 kg/ha) and P_2_O_5 (_40 kg/ha). The pod borer (*Helicoverpa armigera*) was effectively controlled by spraying 0.2% Spinosad at 30, 45, and 60 days after sowing (DAS), respectively. The average daily minimum and maximum temperature were 27.7 °C and 13.1 °C respectively. The total annual rainfall ranged from 0.2 mm to 55.8 mm with a mean of about 5.7 mm with the mean relative humidity (75%).

During the *rabi* 2019–2020, 15–20 seeds harvested from each 183 F_2_ plants were sown in each row of 3 m length and thus each row represented the F_2:3_ generation, an advancement of progenies of a single F_2_ plant. The inheritance pattern observed for stem growth habit in F_2_ was validated in F_3_. Each progeny in F_2:3_ consisted of 15–20 plants. The crop husbandry, as well as protection strategies remained the same as those of the preceding season. The mean daily minimum and maximum temperatures were 25 °C and 37.8 °C, respectively. The total annual rainfall during the season ranged from 0.2 mm to 66 mm with a mean of about 1.643 mm.

### Genetics of stem growth habit

F_1_ and F_2_ plants along with parental genotypes were screened for the trait stem growth habit for inheritance studies. Observations on stem growth habit during flowering along with maximum pod formation stage were recorded on 5 plants of the parents, F_1_ plants as well as all individuals of the F_2_ population. Two different stem elongation patterns could be seen in F_2_ of the IDT × SDT cross. All F_2_ plants with elongated flowering offshoots that ended with the vegetative elongation phase were categorized as indeterminate, plants like those of indeterminate varieties with continuous flowering offshoots but ended with a flower bud or fully opened flower were termed as semi-determinate^9^. The expected values were calculated based on the Mendelian ratio, corresponding to the observed values for indeterminate: semi-determinate plants. To assess the goodness of fit, the deviations of these were analyzed through the chi-square (χ^2^) test.

During the *rabi* 2019–2020, the inheritance pattern observed for stem growth habit in F_2_ was validated in F_2:3_. For stem growth habit, both indeterminate and semi-determinate plants phenotyped in F_2_ were identified and their offsprings were examined along with their corresponding parents. At the stage of maximum flowering and pod formation, each and every offspring was noticed for stem growth habit on an individual plant basis and were classified as non-segregating vs segregating for stem growth habit.

### Isolation of genomic DNA

The young and tender leaf samples from both the parents as well as F_2_ individual plants were collected and DNA was extracted using the procedure of CTAB method^22^ with minor alteration. The DNA was purified using 3 μl RNase (10 μg/μl) and incubated for 30 min at 37 °C. Purified DNA was quantified on agarose gel of 0.8% concentration along with Hind III-cut λ DNA as standard. The concentration of DNA in an individual sample was identified based on the intensity of the bands in the λ DNA ladder. DNA samples were diluted with TE buffer to prepare a working solution with the 25 ng/μl concentration followed by storing at 4 °C.

### PCR amplification

PCR for the specific SSR marker analysis was done in 10 μl reaction volume. The reaction mixture of 10 μl was made by adding 1 μl of 25 ng/μl template DNA, 1 μl of forward primer, 1 μl of reverse primer, 5 μl master mix, and 2 μl nuclease-free water. All the primers were amplified using touchdown PCR Thermocycler from the “Applied Bio System model “Veriti”. The amplification was conducted for 5 min with an initial denaturation at 94 °C followed by a two-step' touch-down' process. The first stage had 18 cycles: denaturation for 30 s ecs at 94 °C, annealing for 1 min at 52–65 °C and extension for 1 min at 72 °C. The second step was set for 20 cycles: denaturation for 30 s at 94 °C, annealing for 1 min at 55 °C and extension for 1 min at 72 °C.

### Gel electrophoresis and visualization of amplicons

Amplified PCR products were separated on 3 per cent agarose media. Using 1.0 × TBE buffer, the amplified products were separated on a horizontal electrophoresis platform for 3–4 h at 120 V. The gels were stained with ethidium bromide (10 mg/ml) and visualized using Gel Documentation (Alpha Imager 2200, Alpha Innotech Corporation, USA) system. For each marker loci, amplicons were graded as alleles. Manual scoring of the alleles was performed and their sizes (bp) were determined by comparison with the ladder of 100 bp DNA.

### Parental polymorphic survey and bulked segregant analysis

Parental polymorphism between BG362 and BG3078-1 was examined using 245 SSR markers^23–32^ Supplementary Table S1. The SSR markers were synthesized by the Integrated DNA Technologies, Inc., 1710 Commercial Park, Coralville, Iowa 52241, USA. The parental polymorphic markers were used for the study of BSA as described by Michelmore et al., (1991). BSA^33^ on stem growth habit was conducted to recognize the molecular markers that were putatively associated with stem growth habit. The BSA was done for two bulks (B1 and B2) of 20 plants for each extreme phenotype for indeterminate forms and semi-determinate types from the individuals in F_2_. The two bulks were screened along with parents using polymorphic SSRs. Amplified products were run on 3% agarose gel. The bands for respective BSA polymorphic markers were verified for consistency by repeating the reactions twice .

## Supplementary Information

Supplementary Tables.

## Data Availability

All data generated or analysed during this study are included in this published article (and its Supplementary Information files).

## References

[1] Varshney RK (2013). Draft genome sequence of chickpea (*Cicer arietinum* L.) provides a resource for trait improvement. Nat. Biotechnol..

[2] Bradley D, Ratcliffe O, Vincent C, Carpenter R, Coen E (1997). Inflorescence commitment and architecture in Arabidopsis. Science.

[3] Tian Z (2017). Artificial selection for determinate growth habit in soybean. Proc. Nat. Acad. Sci. USA.

[4] Bernard RL (1972). Two genes affecting stem termination in soybeans. Crop Sci..

[5] Reinhardt D, Kuhlemeier C (2002). Plant architecture. EMBO Rep..

[6] Huyghe C (1998). Genetics and genetic modifications of plant architecture in grain legumes: A review. Agronomie.

[7] Williams JH, Saxena NP (1991). The use of non destructive measurement and physiological models of yield determination to investigate factors determining differences in seed yield between genotypes of “desi” chickpeas (*Cicer arietinum*). Ann. Appl. Biol..

[8] Renu K-C, Sinha SK (1990). What limits the yield of pulses? Plant processes or plant type. Proc. Int. Congr. Plant Physiol..

[9] Hegde VS (2011). Morphology and genetics of a new found determinate genotype in chickpea. Euphytica.

[10] Shamsuzzaman KM, Gibson AH, Oram RN, Shaikh MAQ (2002). Assimilation and partitioning of dry matter and nitrogen in Hyprosola, a more determinate mutant of chickpea, and in its parental cultivar. Field Crops Res..

[11] Ambika. Genetic analysis of yield traits and identification of markers for stem growth habit using bulked segregant analysis in chickpea (*Cicer arietinum* L.). M.Sc. Agri. Thesis, 1–44 (ICAR-Indian Agricultural Research Institute, 2020).

[12] Parida SK (2015). Development of genome-wide informative simple sequence repeat markers for large scale genotyping applications in chickpea and development of web resource. Front. Plant Sci..

[13] Nayak, S.N. Identification of QTLs and genes for drought tolerance using linkage mapping and association mapping approaches in chickpea (*Cicer arietinum*) Ph.D. thesis (Osmania University, 2010).

[14] Mallikarjuna BP (2017). Molecular mapping of flowering time major genes and QTLs in chickpea (*Cicer arietinum* L.). Front. Plant Sci..

[15] Harshavardhana YS (2019). Genetics of semi-determinacy and identification of molecular marker linked to *Dt1* locus in chickpea (*Cicer arietinum* L.). Indian J. Genet..

[16] Van Rheenen HA, Pundir RPS, Miranda JH (1994). Induction and inheritance of determinate growth habit in chickpea (*Cicer arietinum* L.). Euphytica.

[17] Filippetti A (1986). Inheritance of determinate growth habit induced in *Vicia faba* major by ethyl methane sulphate (EMS). Faba Bean Inf. Serv..

[18] Kapoor RK, Gupta SC (1991). Inheritance of growth habit in pigeonpea. Crop Sci..

[19] Waldia RS, Singh VP (1987). Inheritance of dwarfing genes in pigeonpea. Euphytica.

[20] Vicente D (2016). Mapping and validation of molecular markers of genes *Dt1* and *Dt2* to determine the type of stem growth in soybean. Acta Sci. Agron..

[21] Saxena RK (2017). Characterization and mapping of *Dt1* locus which co-segregates with *CcTFL1* for growth habit in pigeonpea. Theor. Appl. Genet..

[22] Murray MG, Thompson WF (1980). Rapid isolation of high molecular weight plant DNA. Nucleic acids Res..

[23] Agarwal G (2014). Identification of a non-redundant set of 202 in silico SSR markers and applicability of a select set in chickpea (*Cicer arietinum* L.). Euphytica.

[24] Bhardwaj J (2014). In silico development and validation of EST derived new SSR markers for drought tolerance in *Cicer arietinum* L. Indian J. Genet..

[25] Buhariwalla HK, Jayashree B, Eshwar K, Crouch JH (2005). Development of ESTs from chickpea roots and their use in diversity analysis of the Cicer genus. BMC Plant Biol..

[26] Gaur R (2011). Advancing the STMS genomic resources for defining new locations on the intraspecific genetic linkage map of chickpea (*Cicer arietinum* L.). BMC Genomics.

[27] Hüttel B (1999). Sequence-tagged microsatellite site markers for chickpea (*Cicer arietinum* L.). Genome.

[28] Jafari N, Behroozi R, Bagheri A, Moshtaghi N (2013). Determination of Genetic diversity of cultivated chickpea (*Cicer arietinum* .L) using Medicago truncatula EST-SSRs. J. Plant Mol. Breed..

[29] Lichtenzveig J, Scheuring C, Dodge J, Abbo S, Zhang HB (2005). Construction of BAC and BIBAC libraries and their applications for generation of SSR markers for genome analysis of chickpea, *Cicer arietinum* L. Theor. Appl. Genet..

[30] Nayak SN (2010). Integration of novel SSR and gene-based SNP marker loci in the chickpea genetic map and establishment of new anchor points with *Medicago truncatula* genome. Theor. Appl. Genet..

[31] Varshney RK (2019). A comprehensive resource of drought-and salinity-responsive ESTs for gene discovery and marker development in chickpea (*Cicer arietinum* L.). BMC Genomics.

[32] Winter P (1999). Characterization and mapping of sequence-tagged microsatellite sites in the chickpea (*Cicer arietinum* L.) genome. Mol. Gener. Genet. MGG..

[33] Michelmore RW, Paran I, Kesseli RV (1991). Identification of markers linked to disease-resistance genes by bulked segregant analysis: a rapid method to detect markers in specific genomic regions by using segregating populations. Proc. Nat. Aca. Sci. USA.

