# Supplementary material for: Unraveling genetics of semi-determinacy and identification of markers for indeterminate stem growth habit in chickpea (Cicer arietinum L.)
Source: Sci Rep. 2021 Nov 8;11:21837. doi: 10.1038/s41598-021-01464-3 (PMC8575898; doi:10.1038/s41598-021-01464-3)
Supplement: Supplementary file 1 — Supplementary Tables. [file 41598_2021_1464_MOESM1_ESM.docx]

**Supplementary Table S1: List and details of markers used in this study**

| Sl. No. | Marker Name | Forward Primer | Reverse Primer | Reference |
| --- | --- | --- | --- | --- |
| 1. | AGLC101 | TGTCCAAAATTGGGATCAGAGA | AGAACGACTTCAGCAGCAGCA | Buhariwalla HK *et al*., 2005 |
| 2. | AGLC15 | ACTGATCAAGGTCTCTTCTAGACA | CCCAACAAACTGGACAAAGCAGA | Buhariwalla HK *et al*., 2005 |
| 3. | AGLC20 | AATGGTGATTCGTCAGTCGCCTA | CTGTCTGAAGAAAGTGAACGAA | Buhariwalla HK *et al*., 2005 |
| 4. | AGLC28 | GCTAAACCTTAGAGCAATGACTCA | CCTTGCTTGTGCCTTATCTTCCA | Buhariwalla HK *et al*., 2005 |
| 5. | AGLC29 | TCTTCAACACCTCCATCTAACCTA | GACATGAAACCAAAGCATCACA | Buhariwalla HK *et al*., 2005 |
| 6. | AGLC34 | CTTTACCAAAACCACCTTCACCAA | TCTCTCTCTCTCTCTTCTGTTCCA | Buhariwalla HK *et al*., 2005 |
| 7. | AGLC39 | GCCGAGGTACACTTTACCAA | TCCTCACACTTCAGGTTCAACGA | Buhariwalla HK *et al*., 2005 |
| 8. | AGLC48 | TGCCCAACGGTTTCTTTTACCA | TCAGAGATACTCGCCCACCAA | Buhariwalla HK *et al*., 2005 |
| 9. | AGLC52 | CGATCAAGAACCCAGTTTTGCAA | AAGATCGACAGGCGATCTGGTA | Buhariwalla HK *et al*., 2005 |
| 10. | AGLC53 | CACTCTCCGTTCCGGTTCCA | CTGTCCATGCCCTTGTCCA | Buhariwalla HK *et al*., 2005 |
| 11. | AGLC55 | CAGGTCGCGTTGTTGCA | GGCCGAGGTACACTTTTCCA | Buhariwalla HK *et al*., 2005 |
| 12. | AGLC60 | CATGTTTTCTACCCTCACAATGCA | TACTCACTTGTTGTTCCAGACA | Buhariwalla HK *et al*., 2005 |
| 13. | AGLC66 | CCACAAAGGACGACAACAACGA | CCCAACACGAACCACACGA | Buhariwalla HK *et al*., 2005 |
| 14. | AGLC72 | TTTAATTACGCGGTTTCCACGA | GAAGACTTGAGACATGGGCACA | Buhariwalla HK *et al*., 2005 |
| 15. | AGLC74 | CGTGGGATTGAAAAAGTTGCTA | CACTACCAGCCAAAGCACTCA | Buhariwalla HK *et al*., 2005 |
| 16. | AGLC76 | CATGAGTGGTAGTGGGAGTGGA | GTTCGTTTGAGTCGTTTACTGGAA | Buhariwalla HK *et al*., 2005 |
| 17. | AGLC8 | CAAACTCCTCAATAGCAGGCACA | GCTGTATCGGAGAGTGGTCAGA | Buhariwalla HK *et al*., 2005 |
| 18. | AGLC83 | TCTTCCGATCCTAAGAAAGAGCAA | ACCAATATGGAGAGCACCAGTCA | Buhariwalla HK *et al*., 2005 |
| 19. | AGLC88 | ACTTGGGCGTTCAAAAATCTCA | CCATTACGATCAAAGAGCTCAGGA | Buhariwalla HK *et al*., 2005 |
| 20. | AGLC9 | ACTCCTGTAGTGGCATATCTTCGA | TGGTCCATTTATGCCGCTGGTA | Buhariwalla HK *et al*., 2005 |
| 21. | AGLC94 | CCAACTTCCCTCATTCTTATTCCA | ACCAATTCCAAATTTCCAGCTCGA | Buhariwalla HK *et al*., 2005 |
| 22. | AGLC98 | CTCTTTCTTTCCCTCTAGTTTCCA | CGGCGAACTCGTGTTTGCTA | Buhariwalla HK *et al*., 2005 |
| 23. | Ca_GpSSR00002 | ATCGCGTTAAATACTTGGACT | CATATGTGGAATGGACTATGC | Parida SK *et al*., 2015 |
| 24. | Ca_GpSSR00003 | TGAATGGAGTAAATGATTACTTG | TCGCATATAGACGGATAATTT | Parida SK *et al*., 2015 |
| 25. | Ca_GpSSR00028 | ATCGCGTTAAATACTTGGACT | CATATGTGGAATGGACTATGC | Parida SK *et al*., 2015 |
| 26. | Ca_GpSSR00038 | CTGTTACGTGCAATGGATGC | TCGGTATGACACAAAAATGTGA | Parida SK *et al*., 2015 |
| 27. | Ca_GpSSR00040 | ATCGCGTTAAATACTTGGACT | CATATGTGGAATGGACTATGC | Parida SK *et al*., 2015 |
| 28. | Ca_GpSSR00045 | ATCGCGTTAAATACTTGGACT | CATATGTGGAATGGACTATGC | Parida SK *et al*., 2015 |
| 29. | Ca_GpSSR00056 | ATCGCGTTAAATACTTGGACT | CATATGTGGAATGGACTATGC | Parida SK *et al*., 2015 |
| 30. | Ca_GpSSR00081 | TCATGCAACAGACGAATTGA | AACATGTTATAAAACGTAAGGCG | Parida SK *et al*., 2015 |
| 31. | Ca_GpSSR00114 | TGTGACAAAACAAATAACGTGTCA | CGTCAAAGTTAACGCAGTTGAG | Parida SK *et al*., 2015 |
| 32. | Ca_GpSSR00128 | ATCGCGTTAAATACTTGGACT | CATATGTGGAATGGACTATGC | Parida SK *et al*., 2015 |
| 33. | Ca_GpSSR00173 | TGTCACAAACATGTAAGACAGTTGTT | AAGATTTGGCTGGCTTCATC | Parida SK *et al*., 2015 |
| 34. | Ca_GpSSR00195 | ACAATTCCACTTAATCTTTGC | AATTAGCCTACAGACACACACA | Parida SK *et al*., 2015 |
| 35. | Ca_GpSSR00227 | ATTTGCACTCTTTGACTTTGA | TCAAATGTTTAACTCGTGGAC | Parida SK *et al*., 2015 |
| 36. | Ca_GpSSR00231 | ATCTGGGGCCAAAAAGTAGG | CATTCAGCGACAAAAGGTCA | Parida SK *et al*., 2015 |
| 37. | Ca_GpSSR00296 | TGATGTTCTCTGTCTTTCCTT | TCCATATGCAAGAGTAAAAGC | Parida SK *et al*., 2015 |
| 38. | Ca_GpSSR00297 | TCTGGCAGATGAAGATGTAAC | TAACAGACCAAAAACTCCAAA | Parida SK *et al*., 2015 |
| 39. | Ca_GpSSR00318 | TGGCCCAATATTCAGATTCC | TCACCAAACCAGGTACTGCTC | Parida SK *et al*., 2015 |
| 40. | Ca_GpSSR00343 | AATTTACCGCTGAAGAGTTTC | AGGGTAAACTTTAAACTCAAATG | Parida SK *et al*., 2015 |
| 41. | Ca_GpSSR00407 | GAGCACCAATTAAAGAGGGC | GGCATTTAAAACATAAATCCCAA | Parida SK *et al*., 2015 |
| 42. | Ca_GpSSR00458 | TCAAAGGCAATTTTGATTGAA | TTTTTGATAGTCGCAGGACCTT | Parida SK *et al*., 2015 |
| 43. | Ca_GpSSR00459 | CAAATATGCAATAACCTTCCA | AAAAATCTATTTGGTGCTGGT | Parida SK *et al*., 2015 |
| 44. | Ca_GpSSR00477 | CAAATATGCAATAACCTTCCA | AAAAATCTATTTGGTGCTGGT | Parida SK *et al*., 2015 |
| 45. | Ca_GpSSR00483 | CGAACACTGATGCCAAACAA | AAAATGTATTTTATGAGTTTGGCCTAT | Parida SK *et al*., 2015 |
| 46. | Ca_GpSSR00487 | TGAAATTCAGCAACGAAATGA | CCCAGTAACCGCTCTCTCTG | Parida SK *et al*., 2015 |
| 47. | Ca_GpSSR00504 | TCACTTTTACATTACACACAAGGATT | CATGTCTTTTGATATACTACCTCCACA | Parida SK *et al*., 2015 |
| 48. | Ca_GpSSR00512 | TTAAAGACCCTTTTTGGAATC | TCAATCTTTTACTCTCGCAAT | Parida SK *et al*., 2015 |
| 49. | Ca_GpSSR00521 | AATGACGCAGGTTCAACACA | ATGTCATTATGTTGCATTAAGATCTAC | Parida SK *et al*., 2015 |
| 50. | Ca_GpSSR00527 | TCTTCACGCTCCCTACAAAA | TGAGCGTTATAGGGGAGAAAAA | Parida SK *et al*., 2015 |
| 51. | Ca_GpSSR00529 | CTACGTTTCACAATATTTTCGTT | ACCCCTTTTCTCTTTCTTCTA | Parida SK *et al*., 2015 |
| 52. | Ca_GpSSR00535 | AAAATGGTAGGTCATTCATTACT | TACTCTTTCCGCCACAATAA | Parida SK *et al*., 2015 |
| 53. | Ca_GpSSR00536 | TTATCCCGTAAGCCTCGCTA | CCACGTCGGTTCTCTTTGAT | Parida SK *et al*., 2015 |
| 54. | Ca_GpSSR00537 | AGAAAATCAGAAGTTGCATT | AAAGTTTGGTGGTGAGTTTTT | Parida SK *et al*., 2015 |
| 55. | Ca_GpSSR00538 | AAAATAAAAGCTAACGGTCA | TCGAAGTTGTGTCAAACTGTA | Parida SK *et al*., 2015 |
| 56. | Ca_GpSSR00543 | ATCATTTCCTTCGGTTCATC | TTCTTTTCTTCCTAAAAGGACA | Parida SK *et al*., 2015 |
| 57. | Ca_GpSSR00552 | ACCATTTATGAATTACGGTCA | CACACACACATTCACTCTCAA | Parida SK *et al*., 2015 |
| 58. | Ca_GpSSR00556 | AGAAAATCAGAAGTTGCATT | AAAGTTTGGTGGTGAGTTTTT | Parida SK *et al*., 2015 |
| 59. | Ca_GpSSR00560 | GCCTAAAATCATAAAACCAAAG | ATGCAAGGGTTATTTTATGTC | Parida SK *et al*., 2015 |
| 60. | Ca_GpSSR00568 | CATTCCTTCAGCATTTCTTTT | CGTAGAATTTACCGTTGTTCT | Parida SK *et al*., 2015 |
| 61. | Ca_GpSSR00578 | CCAAGCTATAGTGCAATCATCGT | AGGAGGCCTTTGAAAAATCA | Parida SK *et al*., 2015 |
| 62. | Ca_GpSSR00716 | AGAAAATCAGAAGTTGCATT | AAAGTTTGGTGGTGAGTTTTT | Parida SK *et al*., 2015 |
| 63. | Ca_GpSSR00728 | TGCCCTTTTAAGGTTTGTTTG | TGATTTCTCATTTTAAACGGTTTTT | Parida SK *et al*., 2015 |
| 64. | Ca_GpSSR00736 | AGAAAATCAGAAGTTGCATT | AAAGTTTGGTGGTGAGTTTTT | Parida SK *et al*., 2015 |
| 65. | Ca_GpSSR00741 | GCCTAAAATCATAAAACCAAAG | ATGCAAGGGTTATTTTATGTC | Parida SK *et al*., 2015 |
| 66. | Ca_GpSSR00834 | AACGCTTTCCTACCAATTTTT | GGAGGAAGTCGTTAACAAAAGA | Parida SK *et al*., 2015 |
| 67. | Ca_GpSSR00931 | TCATCTGATCACTAATACACCAA | GAAGAAAAAGAACCATTTGACT | Parida SK *et al*., 2015 |
| 68. | Ca_GpSSR00943 | CTTGGTGTGATCTTCACGGA | AGACAAAAACGGGCTAGTTAAA | Parida SK *et al*., 2015 |
| 69. | Ca_GpSSR00954 | CTTCTTTACATCTGCTCTCCA | GCGCAAAATTCCTTTTAGTAT | Parida SK *et al*., 2015 |
| 70. | Ca_GpSSR00955 | GTCCCAAAGCAATTTATTATC | CACTGCCACCGTATTATTAAA | Parida SK *et al*., 2015 |
| 71. | Ca_GpSSR00972 | TTTTTGTTACAACCATTTTTGGAT | GAAGCTATTTTGAATTCTTTTGTTAAA | Parida SK *et al*., 2015 |
| 72. | Ca_GpSSR00976 | TGGTTTTTAACAAAAATATGTGGC | TCCCTCTTAAATTGATGGCAA | Parida SK *et al*., 2015 |
| 73. | Ca_GpSSR01024 | AACCACATTTGTTGTCGCTG | CAAAATAAATGAAGGAGAGAATAAAGC | Parida SK *et al*., 2015 |
| 74. | Ca_GpSSR01067 | GAGCTCAATAATTGGATTAGATTTATG | TGGTACAAAATACCAGGATCAA | Parida SK *et al*., 2015 |
| 75. | Ca_GpSSR01116 | ATTTGCACTCTTTGACTTTGA | TCAAATGTTTAACTCGTGGAC | Parida SK *et al*., 2015 |
| 76. | Ca_GpSSR01156 | ATTTGCACTCTTTGACTTTGA | TCAAATGTTTAACTCGTGGAC | Parida SK *et al*., 2015 |
| 77. | Ca_GpSSR01194 | ATTTGCACTCTTTGACTTTGA | TCAAATGTTTAACTCGTGGAC | Parida SK *et al*., 2015 |
| 78. | Ca_GpSSR01239 | GACGATTCTCCATAACTGTGA | ACAAAGGTTGTGTTTCCTAAA | Parida SK *et al*., 2015 |
| 79. | Ca_GpSSR01240 | AGCACTTGTTAACGTTTTCTT | GTCAATCTATTGTGATGTTTTTG | Parida SK *et al*., 2015 |
| 80. | Ca_GpSSR01242 | ATTTGCACTCTTTGACTTTGA | TCAAATGTTTAACTCGTGGAC | Parida SK *et al*., 2015 |
| 81. | Ca_GpSSR01289 | AGCGAAATGTATTTCAGTGAG | CTTTCATTCTTCTCTCCCATT | Parida SK *et al*., 2015 |
| 82. | Ca_GpSSR01481 | ACCTCGATAAACAGTACAGACC | TGAATTTGAGTGTTAGCGTTT | Parida SK *et al*., 2015 |
| 83. | Ca_GpSSR01487 | AATCCACAAACACAACAAAAC | CCAATCAACTCATTGAGACC | Parida SK *et al*., 2015 |
| 84. | Ca_GpSSR01488 | AGCCCCTCTTACTCTCACATA | TTGTATGTTTGCTCCGTGTAT | Parida SK *et al*., 2015 |
| 85. | Ca_GpSSR01498 | AGCCCCTCTTACTCTCACATA | TTGTATGTTTGCTCCGTGTAT | Parida SK *et al*., 2015 |
| 86. | Ca_GpSSR01503 | AATCTGACCATAAGTTGACGA | AGAACTATTACTGCATACCTCCA | Parida SK *et al*., 2015 |
| 87. | Ca_GpSSR01504 | AGCCCCTCTTACTCTCACATA | TTGTATGTTTGCTCCGTGTAT | Parida SK *et al*., 2015 |
| 88. | Ca_GpSSR01505 | GGGAGGGATGGAACCTTTTA | TCATTTGTGTGGTTGCTCTTG | Parida SK *et al*., 2015 |
| 89. | Ca_GpSSR01543 | AATCCACAAACACAACAAAAC | CCAATCAACTCATTGAGACC | Parida SK *et al*., 2015 |
| 90. | Ca_GpSSR01555 | AATCCACAAACACAACAAAAC | CCAATCAACTCATTGAGACC | Parida SK *et al*., 2015 |
| 91. | Ca_GpSSR01657 | GACAAGCCTCCGTGTGATTT | ATCGACGTCGCTAAATGCTT | Parida SK *et al*., 2015 |
| 92. | Ca_GpSSR01758 | GCATGTTGATTTTGATAAGGA | CTTCTTCTGATGCCATGACT | Parida SK *et al*., 2015 |
| 93. | CakTSSR00118 | GTTCACCACAGAATTCATCAT | ACGATTTCCGATTCATCTTA | Agarwal G *et al*., 2015 |
| 94. | CakTSSR00621 | TTCTCTCTCGTCTCTGGAGTT | TCCCTCGCAACTAATATAACC | Agarwal G *et al*., 2015 |
| 95. | CakTSSR00729 | TCGTAGACACGAAAATCTGTT | AACCTTGATCAACATCTGGTA | Agarwal G *et al*., 2015 |
| 96. | CakTSSR01526 | GATGCCATGTTACAACAACTT | TAGTTCATTCAAAGCCTCTCA | Agarwal G *et al*., 2015 |
| 97. | CakTSSR02667 | CTATGACAAAGTGGCATGATT | ATCCACTTATCATTTGACGTG | Agarwal G *et al*., 2015 |
| 98. | CakTSSR03248 | CTAAAGAATGGAATTGGGATT | CTCGTTTGTTTGCTCTATTGT | Agarwal G *et al*., 2015 |
| 99. | CakTSSR03970 | TGAGGTTGAGAATTTGAGTGT | CTCACTTCTCATCACCATCAT | Agarwal G *et al*., 2015 |
| 100. | CakTSSR04407 | TTGAACGATGATCGATAGAAG | GGTGATCCAACCTAGAAGAAC | Agarwal G *et al*., 2015 |
| 101. | CaSTMS 10 | ATAACAAAAAGATATCTCATCGACA | AACAATATACAATAAATAACCAAGT | Huttel B *et al*., 1998 |
| 102. | CaSTMS 14 | TTGTGTTTCTCCTAATATTCTATTAGC | GAATATGAATAACGTTACA | Huttel B *et al*., 1998 |
| 103. | CaSTMS 16 | ATCTTAGAATATCTCTTATTA | ATTACAAAGGACTCAACA | Huttel B *et al*., 1998 |
| 104. | CaSTMS 19 | TGAAGCTGGGGGTTCCTTG | TCAATTGAGTCGCGACGAGAG | Huttel B *et al*., 1998 |
| 105. | CaSTMS 22 | CTCTTCCTCCTCGAGATC | ATAGATACAATACTCTGTGAGTTGG | Huttel B *et al*., 1998 |
| 106. | CaSTMS 25 | TACACTACTGCTATTGATATGTGGT | GACAATGCCTTTTTCCTT | Huttel B *et al*., 1998 |
| 107. | CaSTMS 28 | CCCTTCTAGTGATATTTTG | AAATGTGTTTTATGGAATAAGTCAT | Huttel B *et al*., 1998 |
| 108. | CaSTMS12 | GTATTTGTTACTGCATATACTTAATTA | TATTTACTAGGTAAATCCTATTTATTG | Huttel B *et al*., 1998 |
| 109. | CaSTMS15 | CTTGTGAATTCATATTTACTTATAGAT | ATCCGTAATTTAAGGTAGGTTAAAATA | Huttel B *et al*., 1998 |
| 110. | CaSTMS2 | ATTTTACTTTACTACTTTTTTCCTTTC | AATAAATGGAGTGTAAATTTCATGTA | Huttel B *et al*., 1998 |
| 111. | ES-EST-SSR 108 | GGCTCTTTTCCCCTATGCTC | CCAACATTGCAAGTCCTGAA | Jafari N *et al*., 2013 |
| 112. | EV-EST-SSR 18 | TGGGAACTCCCTTTTCACAC | ATGGCAGTGCATCGAACATA | Jafari N *et al*., 2013 |
| 113. | EY-EST-SSR 1 | CGGAAGCTTTTGCTTCTGTT | GCGAAATACGTGCAAGATCA | Jafari N *et al*., 2013 |
| 114. | GA11 | GTTGAGCAACAAAGCCACAA | TTCTTGTCTGGTTGTGTGAGC | Winter P *et al*., 1999 |
| 115. | GA16 | CACCTCGTACCATGGTTTCTG | TAAATTTCATCCTCTCCGGC | Winter P *et al*., 1999 |
| 116. | GA2 | TGCATTGGAAATACAGCATGA | AATTTTGGTTCGCCACAAAC | Winter P *et al*., 1999 |
| 117. | GA22 | ATGAGTATCAAGCCAACCTGA | GTCCCAACAATTTCTTACATGC | Winter P *et al*., 1999 |
| 118. | GA26 | GATGCTCAAGACATCTGCCA | TCATACTCAACAAATTCATTTCCC | Winter P *et al*., 1999 |
| 119. | GA33 | CAAGCACAATCTTCGTCCAA | CTCTCCATTTGCCTCCTTCA | Winter P *et al*., 1999 |
| 120. | GA8 | GCTCTAAAGGGAAGGCGATT | AACCACCAAAGTTCCCCAG | Winter P *et al*., 1999 |
| 121. | GAA40 | TTGACGCAGAGAACTCTCAA | ATTGGTGTGATGGGTGGATT | Winter P *et al*., 1999 |
| 122. | GAA43 | TGATCGGAGAGAGAGGAGGA | CGTTGATCCACTGCGATAGT | Winter P *et al*., 1999 |
| 123. | H1A18 | CTTTCCTTGGTCTTTGTCTTCAT | TTGTAATTAATGAACATGAGTTAAGAGT | Lichtenzveig L *et al*., 2005 |
| 124. | H1B09 | GGTTTCATGACCTGCACCTA | AAGAACCGAAAACACTTGTGA | Lichtenzveig L *et al*., 2005 |
| 125. | H1D221 | TTCTAGAAACTGTCGACTGATAG | ACTTAATCCATGAAATTTGTTTT | Lichtenzveig L *et al*., 2005 |
| 126. | H1F05 | ATAACTCAAATCGTTTCACAAGA | AAACCCCTTTTTATTTTCAATTT | Lichtenzveig L *et al*., 2005 |
| 127. | H1F14 | GAGAGAGAGGAAGGGAAACG | TCCTAACTTGCTCCTTAACCTTG | Lichtenzveig L *et al*., 2005 |
| 128. | H1H13 | TTCCTTTATCGCACCCTTCT | CCAGAGAAAGAGCAGCTACG | Lichtenzveig L *et al*., 2005 |
| 129. | H1H14 | AGGAATGATTTTCCGGTGAG | TCGAGTGAACAAACCACACA | Lichtenzveig L *et al*., 2005 |
| 130. | H1P17 | TGCCTCCCACTTACATTAGG | TTGCACGAAGACCATTAGAA | Lichtenzveig L *et al*., 2005 |
| 131. | H2I20 | TGTTTTGCTCATCTGTTAAATCAA | AGCATGCCTCTGATGAATAGTAAC | Lichtenzveig L *et al*., 2005 |
| 132. | H2J20 | TCAAAGAGATAAACACTTGTTCAAAA | GGAACTTTAAGTGTTTGCTTCTAGC | Lichtenzveig L *et al*., 2005 |
| 133. | H3A10 | TTTAAGGCTTCAGGTATTGATTTCT | TCACACATGCCAACTTAAAATAAAA | Lichtenzveig L *et al*., 2005 |
| 134. | H3C06 | AATTTCGTGAATCATTAAAAATAGAGG | CACATGACTATCTAGACATTTTATTTATC | Lichtenzveig L *et al*., 2005 |
| 135. | H3DO5 | AGACGTGTTCCCTTTCTTTTAACTA | GCCGACACAAAGTTTATGATTTT | Lichtenzveig L *et al*., 2005 |
| 136. | H3E04 | GATTTAACGTGTCGCGTCTTC | GCCTTATGTGTTTTCCTTAGTGATT | Lichtenzveig L *et al*., 2005 |
| 137. | H3EO52 | TAGACCCTTGCTTCTTGTTCCT | AATCTTGTTGGTTCTTTGGTCA | Lichtenzveig L *et al*., 2005 |
| 138. | H3F08 | AAACACCCGTGATTCTCTAAAGTT | TGACACCTAATTTTATTCGGTTTTT | Lichtenzveig L *et al*., 2005 |
| 139. | H3GO6 | AATTCAAGGACGAATTTTTATAACG | GGAAGGAAAAATGAATTAAAAATGA | Lichtenzveig L *et al*., 2005 |
| 140. | H3H07 | GAGGCATAGTACCTCAATTTTATTCA | AAGAAAGACAGGTTATCTGTGTGGT | Lichtenzveig L *et al*., 2005 |
| 141. | H4A04 | GCAAATTCTCACCATTTCTTTTT | TGTTTTGACGAATGAGAAGTAAAGA | Lichtenzveig L *et al*., 2005 |
| 142. | H4D02 | CAAATCCCTTTTATTTTTCCTTCATA | CTCCCTAAGTAGAACTCACCAATTGTA | Lichtenzveig L *et al*., 2005 |
| 143. | H4D08 | TGTCCTTTATTTCTTAAGCACACAT | GAGATGGATGTTATTGGACTCATC | Lichtenzveig L *et al*., 2005 |
| 144. | H4E09 | TGCTATTTGTACTAGGACTTAAGGAAA | TGTTTAAAGTACCCATTAAAAACGTAA | Lichtenzveig L *et al*., 2005 |
| 145. | H4F03 | CCTTTAACAAACAAAACAAACTTCC | GGTCGTCTGTAACATCCTATATCAA | Lichtenzveig L *et al*., 2005 |
| 146. | H4G01 | ATTGACTTTATGTTGTGATTTTCTTCA | TATCATGCATATTTTCAACTTGTCCTA | Lichtenzveig L *et al*., 2005 |
| 147. | H4G11 | ATCTAAGTGAGCGGCTACTAAATCA | GTAGTCATGCAGCCTATAAAAACAA | Lichtenzveig L *et al*., 2005 |
| 148. | H4H01 | TAGTATTTTTCTTTCATTTCCTTCGTT | TTGCTTTAATTCATTATGGTATATTTATG | Lichtenzveig L *et al*., 2005 |
| 149. | H4H06 | CACAACAGCTCCAACAGATTG | ATGTGCAACTTCAACCACCTAT | Lichtenzveig L *et al*., 2005 |
| 150. | H5A08 | AGGAGAGAAAATGTAACATCCTAAATC | CAAATTGGTTATTGATTACAATTAGGT | Lichtenzveig L *et al*., 2005 |
| 151. | H5B04 | CATAATTTTAAAAGAGGCACGTTAAAT | ATATAAGCAAAAATAAAGATGAGTTGC | Lichtenzveig L *et al*., 2005 |
| 152. | H5E11 | ATTTATTTAGTGGCCTCACTCTCC | GATTTCAAAATCCCTTTGTTCTAGTTA | Lichtenzveig L *et al*., 2005 |
| 153. | H6C07 | CAAAAGTGCAATTAAGCCTACATAATA | CCATTTTGTTTTAACACATATTTAACG | Lichtenzveig L *et al*., 2005 |
| 154. | H6C11 | TTGGGTTATCCAAAGAATAAAATATAA | TTATATGGACTATGTATGTCAATTAAATC | Lichtenzveig L *et al*., 2005 |
| 155. | H6D11 | AAAGATGGGAACTTGAGATGTTG | AATAGCTACTCAAGGCTGAAGAAA | Lichtenzveig L *et al*., 2005 |
| 156. | ICCeM0011 | CACTCTGCAGGCACTCTTTG | CACGTTCATTTCTTCACCCC | Varshney RK *et al*., 2009 |
| 157. | ICCeM0013 | TGTGTCAGCAACTGGAGGAG | GCCTTCTACACAACGGCTTC | Varshney RK *et al*., 2009 |
| 158. | ICCeM0033 | AGCGTGTTCCATTCTGCTCT | TAGGACAATTCGAGGGTTCG | Varshney RK *et al*., 2009 |
| 159. | ICCeM0046 | TCTACGCCTATGGCTTCTCC | CAACGCTCATTCCATTTCCT | Varshney RK *et al*., 2009 |
| 160. | ICCeM0051 | CGTTGGCAGTTCCGTTCTAT | CTCCGCCATCATCATTTCTT | Varshney RK *et al*., 2009 |
| 161. | ICCeM0054 | TTCCGTTTTCAAACCCAAAC | AACAGAGAGCTCCGAATCCA | Varshney RK *et al*., 2009 |
| 162. | ICCeM0055 | GTGTGTGCAGCTGAAAAGGA | AAGAGATGCGCCCATTGTAG | Varshney RK *et al*., 2009 |
| 163. | ICCeM0063 | TTGTTGCAAAGCATCCTTCA | GGAAATTGAGGGAAGAGGGA | Varshney RK *et al*., 2009 |
| 164. | ICCM0003 | AATGGAAGAACGTCAGGGTG | TTCCACTGGGGCAAAATAAG | Nayak SN *et al*., 2010 |
| 165. | ICCM0010b | ACGCCAATTCTTTTGAGCAC | TCAGCACTGGTGGAACCATA | Nayak SN *et al*., 2010 |
| 166. | ICCM0022 | TAAACCGCATTGACGAATGA | TGAATTCGCAAGAATCAAATG | Nayak SN *et al*., 2010 |
| 167. | ICCM0078b | AATCCCAACGGTGAGAGATG | GGACAAGGAGTGGAAGGGA | Nayak SN *et al*., 2010 |
| 168. | ICCM0120a | TGTCTCGATAAGAGTTTGTTATTTTTC | CGTTTTGTTTCATATTCAAACTCG | Nayak SN *et al*., 2010 |
| 169. | ICCM0123a | GGATGGTCTGCTGGAATCAT | AAAGACAACAAAAAGACAATCATGT | Nayak SN *et al*., 2010 |
| 170. | ICCM0160 | TTGCTTGAAACAACCTTTCG | CGGGTACAACCGTAGCAAAT | Nayak SN *et al*., 2010 |
| 171. | ICCM0178 | AGTTTGGGTTTCACCGCCT | GAACGCGCTCTGTTCATAAT | Nayak SN *et al*., 2010 |
| 172. | ICCM0190a | GGGGGATTGTCTGAGTTTCA | AAAAGGCTGGAGACACCTCA | Nayak SN *et al*., 2010 |
| 173. | ICCM0192a | GCTGCCCAAATTTTGACATTA | CCGGGGATCAAATTCTTCTT | Nayak SN *et al*., 2010 |
| 174. | ICCM0199c | TTAGAGGCAAACCAGAACCG | ATCTTGAAGTGGGCAAAACG | Nayak SN *et al*., 2010 |
| 175. | ICCM0202b | TGAACCAGAATCAGAGGCAA | CCAATTTGGTCCGGTTTTTA | Nayak SN *et al*., 2010 |
| 176. | ICCM0224 | ACCACCTTGCTCATCCTCAC | GAGTAGGAGGTGCGAAAACG | Nayak SN *et al*., 2010 |
| 177. | ICCM0229 | TGTCTTATTCCTCCTCCCCC | AGGGGTTTTTGGGTTACCAG | Nayak SN *et al*., 2010 |
| 178. | ICCM0240b | ACCCGAACCCGCAAATAATA | GCAATGAGACTGGGGTTTTC | Nayak SN *et al*., 2010 |
| 179. | ICCM0242a | TGCATTCATCTGTTTCGCTC | GAAAATATTTGTGGTTATCCGATTTT | Nayak SN *et al*., 2010 |
| 180. | ICCM0243c | ACGACGATTCTGGATTTTGG | AGTTTTGGTAGGGGGTCGAG | Nayak SN *et al*., 2010 |
| 181. | ICCM0245 | GCGGCTGGTTTAAGAGTGAG | CCAACACGACCCAAATCAAT | Nayak SN *et al*., 2010 |
| 182. | ICCM0288 | TTATTTTTCGGATCCAACGC | GTGATTTTTGTTCGGCCATT | Nayak SN *et al*., 2010 |
| 183. | ICCM0289 | CAGCCTCCATGGCATAGATAA | TGCTTGAATGAGTGCAACAA | Nayak SN *et al*., 2010 |
| 184. | NCPGR12 | CCTTGTTAGTGTGTATAGGT | GTAATGACCAAGTGAACA | Gaur R *et al*., 2011 |
| 185. | NCPGR21 | TCTACCTCGTTTTTCGTGCC | TTGCTCCTTCAACAAAACCC | Gaur R *et al*., 2011 |
| 186. | NCPGR33 | ACATCTTGAAGTGCCCCAAC | TGCAAGCAGACGGTTACAAG | Gaur R *et al*., 2011 |
| 187. | NCPGR4 | TTACAGCTTGTGCTCAG | AGTCAGATTCTTATCCGA | Gaur R *et al*., 2011 |
| 188. | NCPGR42 | CCCCTAGTAGCAAATATTTTGACC | TTTGAATGCATTTCTTCATAGCA | Gaur R *et al*., 2011 |
| 189. | NCPGR48 | TGGGCTATGAATTAAGATGG | TAATTGATGAGGGAGAGAGCCC | Gaur R *et al*., 2011 |
| 190. | NCPGR57 | CGATGATATTCTCAGCGAAC | TGTATGAAAACACTTTGACTCATT | Gaur R *et al*., 2011 |
| 191. | NCPGR57 | CGATGATATTCTCAGCGAAC | TGTATGAAAACACTTTGACTCATT | Gaur R *et al*., 2011 |
| 192. | NCPGR69 | GACCGAATGTCCATAAATCA | GGAGCTGGAAAAACTACAGC | Gaur R *et al*., 2011 |
| 193. | NCPGR81 | CCGAATGTCCATAAATCAAT | TGTTTGACTGGGATAACTCC | Gaur R *et al*., 2011 |
| 194. | NCPGR93 | CAAAGTTTGTTGCTAGGATTC | GAAGATCTCCGACGATGATA | Gaur R *et al*., 2011 |
| 195. | SVP 11 | TCTCAGTTCCCTCATTCAAC | ATTTCTCCCACCAGTCTTTT | Bhardwaj J *et al*., 2014 |
| 196. | SVP 162 | TCTCAGTTCCCTCATTCAAC | ATTTCTCCCACCAGTCTTTT | Bhardwaj J *et al*., 2014 |
| 197. | SVP 180 | CCCCAAACAACATTATCC | GAACTGGTGGGAAATACAC | Bhardwaj J *et al*., 2014 |
| 198. | SVP 181 | ATATGCAGCCAGCAAAACTG | GGTTCGGATTGTCACTTGCT | Bhardwaj J *et al*., 2014 |
| 199. | SVP 219 | CAAAATCCCACACCACTACT | GTTTCCATAACCACCTGTGT | Bhardwaj J *et al*., 2014 |
| 200. | SVP 221 | GTAGGACCGATGAATATGGA | CTTATTTTACCGACTGCACC | Bhardwaj J *et al*., 2014 |
| 201. | SVP 31 | CTTTTGGCTCCACCCATT | GAGGAAAGGTTATTCGGGTAAA | Bhardwaj J *et al*., 2014 |
| 202. | SVP 311 | GGGTCCAATAATCTGTTGTG | AGCCACATAAACATGGAGA | Bhardwaj J *et al*., 2014 |
| 203. | TA103II | TCTGCAAAAACTATTACGTTAATACCA | TTGTGTGTAATGGA TTGAGTATCTCTT | Winter P *et al*., 1999 |
| 204. | TA11 | CATGCCATAAACTCAATACAATACAAC | TTCATTGAGGACAATGTGTAATTTAAG | Winter P *et al*., 1999 |
| 205. | TA110 | ACACTATAGGTATAGGCATTTAGGCAA | TTCTTTATAAATATCAGACCGGAAAGA | Winter P *et al*., 1999 |
| 206. | TA113 | TCTGCAAAAACTATTACGTTAATACCA | TTGTGTGTAATGGATTGAGTATCTCTT | Winter P *et al*., 1999 |
| 207. | TA116 | AATTCAATGACGAATTTTTATAAGGG | AAAAAGAAAAGGGAAAAGTAGGTTTTA | Winter P *et al*., 1999 |
| 208. | TA117 | GAAAATCCCAAATTTTTCTTCTTCT | AACCTTATTTAAGAATATGAGAAACACA | Winter P *et al*., 1999 |
| 209. | TA118 | ACAAGTCACATGTGTTCTCAATA | GGAAAGGTTAAGAAATTTTACAATAC | Winter P *et al*., 1999 |
| 210. | TA125 | TTGAAATTGAACTGTAACAGAACATAAA | TAGATAGGTGATCACAAGAAGAGAATG | Winter P *et al*., 1999 |
| 211. | TA132 | CGA ATAACTGAGAAAAAGA AATTAG | TTCTAAAACTTCCTTCTACCATTAG | Winter P *et al*., 1999 |
| 212. | TA142 | TGTTAACATTCCCTAATATCAATAACTT | TTCCACAATGTTGTATGTTTTGTAAG | Winter P *et al*., 1999 |
| 213. | TA146 | CTAAGTTTAATATGTTAGTCCTTAAATTAT | ACGAACGCAACATTAATTTTATATT | Winter P *et al*., 1999 |
| 214. | TA176 | ATTTGGCTTAAACCCTCTTC | TTTATGCTTCCTCTTCTTCG | Winter P *et al*., 1999 |
| 215. | TA180 | CATCGTGAATATTGAAGGGT | CGGTAAATAAGTTTCCCTCC | Winter P *et al*., 1999 |
| 216. | TA194 | TTTTTGGCTTATTAGACTGACTT | TTGCCATAAAATACAAAATCC | Winter P *et al*., 1999 |
| 217. | TA2 | AAATGGAAGAAGAATAAAAACGAAAC | TTCCATTCTTTATTATCCATATCACTACA | Winter P *et al*., 1999 |
| 218. | TA200 | TTTCTCCTCTACTATTATGATCACCAG | TTGAGAGGGTTAGAACTCATTATGTTT | Winter P *et al*., 1999 |
| 219. | TA206 | GTCCCACTTCCACTTATAAAGGTT | TAACGTATCTTGCAGATTTCAAATAAA | Winter P *et al*., 1999 |
| 220. | TA22 | TCTCCAACCCTTTAGATTGA | TCGTGTTT ACTGAATGTGGA | Winter P *et al*., 1999 |
| 221. | TA27 | GATAAAATCATTATTGGGTGTCCTTT | TTCAAATAATCTTTCATCAGTCAAATG | Winter P *et al*., 1999 |
| 222. | TA3 | AATCTCAAAATTCCCCAAATT | ATCGAGGAGAGAAGAACCAT | Winter P *et al*., 1999 |
| 223. | TA37 | ACTTACATGAATTATCTTTCTTGGTCC | CGTATTCAAATAATCTTTCATCAGTCA | Winter P *et al*., 1999 |
| 224. | TA42 | ATATCGAAATAAATAACAACAGGATGG | TAGTTGATACTTGGATGATAACCAAAA | Winter P *et al*., 1999 |
| 225. | TA46 | TTTATTGCAATAAAACTCATTTCTTATC | TTCTTTTTGTGTGAAAAAAAAATATAGTGA | Winter P *et al*., 1999 |
| 226. | TA64 | ATATATCGTAACTCATTAATCATCCGC | AAATTGTTGTCATCAAATGGAAAATA | Winter P *et al*., 1999 |
| 227. | TA71 | CGATTTAACACAAAACACAAA | CCTATCCATTGTCATCTCGT | Winter P *et al*., 1999 |
| 228. | TA72 | GAAAGATTTAAAAGATTTTCCACGTTA | TTAGAAGCATATTGTTGGGATAAGAGT | Winter P *et al*., 1999 |
| 229. | TA76s | TCCTCTTCTTCGATATCATCA | CCATTCTATCTTTGGTGCTT | Winter P *et al*., 1999 |
| 230. | TA78 | CGGTAAATAAGTTTCCCTCC | CATCGTGAATATTGAAGGGT | Winter P *et al*., 1999 |
| 231. | TA8 | AAAATTTGCACCCACAAAATATG | CTGAAAA TTATGGCAGGGAAAC | Winter P *et al*., 1999 |
| 232. | TA80 | CGAATTTTTACATCCGTAATG | AATCAATCCATTTTGCATTC | Winter P *et al*., 1999 |
| 233. | TA96 | TGTTTTGGAGAAGAGTGATTC | TGTGCATGCAAATTCTTACT | Winter P *et al*., 1999 |
| 234. | TAA104 | CCCCTAAATTAACAACATAATGG | CGGCTTATGAATTTTTATCATTTACAG | Winter P *et al*., 1999 |
| 235. | TAA58 | CATTGCTTAAGAACCAAAATGCAA | TTTTACATCGACGTGTGC | Winter P *et al*., 1999 |
| 236. | TAASH | GGTAGACGCAAAAGAGTGGG | GCCACATTGACCAGGAATG | Winter P *et al*., 1999 |
| 237. | TR20 | ACCTGCTTGTTTAGCACAAT | CCGCATAGCAATTTATCTTC | Winter P *et al*., 1999 |
| 238. | TR29 | GCCCACTGAAAAATAAAAAG | ATTTGAACCTCAAGTTCTCG | Winter P *et al*., 1999 |
| 239. | TR31 | CTTAATCGCACATTTACTCTAAAATCA | ATCCATTAAAACACGGTTACCTATAAT | Winter P *et al*., 1999 |
| 240. | TR43 | AGGACGAAACTATTCAAGGTAAGTAGA | AATTGAGATGGTATTAAATGGATAACG | Winter P *et al*., 1999 |
| 241. | TR59 | AAAAGGAACCTCAAGTGACA | GAAAATGAGGGAGTGAGATG | Winter P *et al*., 1999 |
| 242. | TR7 | GCATTATTCACCATTTGGAT | TGTGATAATTTTCTAAGTGTTTT | Winter P *et al*., 1999 |
| 243. | TS104 | TCAAGATTGATATTGATTAGATAAAAGC | CTTTATTTACCACTTGCACAACACTAA | Winter P *et al*., 1999 |
| 244. | TS45 | TGACACAAAATTGTCTCTTGT | TGTTCTTAACGTAACTAACCTAA | Winter P *et al*., 1999 |
| 245. | TS72 | CAAACAATCACTAAAAGTATTTGCTCT | AAAAATTGATGGACAAGTGTTATTATG | Winter P *et al*., 1999 |

**Supplementary Table S2: Important characteristics of chickpea genotypes, BG 362 (P_1_) and BG 3078-1 (P_2_) used in this study**

| Sl. No. | Characters | BG 362 (P_1_) | BG 3078-1(P_2_) |
| --- | --- | --- | --- |
| 1. | Stem growth habit | Indeterminate | Semi-determinate |
| 2. | Seed type | Desi | Desi |
| 3. | Leaf type | Normal | Normal |
| 4. | Seed shape | Angular | Angular |
| 5. | Testa texture | Rough | Smooth |
| 6. | Testa colour | Yellow | Light brown |
| 7. | Flower colour | Pink | Pink |
| 8. | Plant growth habit | Semi-erect | Semi-erect |
| 9. | Plant height(cm) | 56.22 | 55.44 |
| 10. | Internodal length(cm) | 2.39 | 1.8 |
| 11. | Days to 50% flowering(days) | 75 | 56.00 |
| 12. | Days to maturity(days) | 135 | 119.23 |
| 13. | Number of pods/plants | 112 | 94.67 |
| 14. | Number of seeds/plants | 103.53 | 103.53 |
| 15. | Number of seeds/pods | 1.36 | 1.36 |
| 16. | 100-seed weight(g) | 23.23 | 20.87 |
| 17. | Seed yield/plant(g) | 21.78 | 19.54 |

**Supplementary Table S3: Descriptive statistics of quantitative characters of F_2_ population**

| Sl. No. | Trait | Mean | | SE | Range | | Variance | SD | CV (%) |
| --- | --- | --- | --- | --- | --- | --- | --- | --- | --- |
|  | | | |  | **Min** | **Max** |  | | |
| 1. | PH (cm) | | 67.16 | 0.84 | 40.5 | 88 | 102.48 | 10.12 | 15.07 |
| 2. | NPPL | | 77.45 | 1.51 | 46 | 110 | 336.45 | 18.34 | 23.68 |
| 3. | NSPL | | 123.98 | 1.71 | 89 | 167 | 434.23 | 20.83 | 16.80 |
| 4. | NSPP | | 1.64 | 0.02 | 1.11 | 2.59 | 0.07 | 0.27 | 16.84 |
| 5. | SY(g) | | 28.52 | 0.44 | 16 | 37.87 | 29.10 | 5.39 | 18.91 |
| 6. | 100-SW(g) | | 23.03 | 0.20 | 16.47 | 28.42 | 6.42 | 2.53 | 11.00 |

PH- Plant height; NPPL- Number of pods per plant; NSPL- Number of seeds per plant; NSPP- Number of seeds per pod; SY- Seed yield per plant; 100 SW- 100-Seed weight

SE-Standard error; SD-Standard deviation; CV-Co-efficient of variation
